# Significance of Ligand-Anchored Polymers for Drug Targeting in the Treatment of Colonic Disorders

**DOI:** 10.3389/fphar.2019.01628

**Published:** 2020-02-25

**Authors:** Pramila Chaubey, Munira Momin, Sujata Sawarkar

**Affiliations:** ^1^ Department of Pharmaceutics, College of Pharmacy, Shaqra University, Al-Dawadmi, Saudi Arabia; ^2^ Department of Pharmaceutics, SVKM’s Dr. Bhanuben Nanavati College of Pharmacy, Mumbai, India

**Keywords:** colon targeted, colorectal cancer, active targeting, ligand anchored, nanocarriers

## Abstract

Treatment of a variety of bowel diseases like Crohn’s disease, ulcerative colitis, colonic cancers, colonic pathologies, and systemic delivery of drugs at the target sites can be done with the help of targeted drug delivery technique. Conventional colon specific drug delivery systems lack specificity and release significant amount of drug prior reaching the target site. Hence, efficient drug delivery system that ensures effective release of the drug at the colon is still a sought after research arena. Ligand anchored therapy is a strong and effective approach to execute drug delivery in selective target cells, for both, diagnostic, as well as therapeutic reasons. Compared to the regular drugs, such ligand anchored therapy provides added benefit of minimum toxicity and few side effects. Discovery of overexpressed receptors on diseased cells, as compared to healthy cells led to the emergence of active drug targeting. Further, drug resistance constitutes one of the major reasons of the failure of chemotherapy and presents a major obstacle for the effective treatment. The reason behind drug resistance is exposure of pathological cells/pathogens to sub-therapeutic levels of drugs due lack of specificity of therapeutics. Active targeting, specifically taken up by the target cells, can warrant exposure of pathological cells/pathogens to high drug load at the target and sparing non-target cells hence minimal damage to normal cells and least chance of drug resistance. Many ligands like antibodies, aptamers, peptides, folate, and transferrin have been discovered in the past few years. The design of nanocarriers can be incorporated with many different functions which enables functions like imaging and triggered intracellular drug release. The present review article focuses on advances in ligand anchored therapy and its significance on the progress of targeted nanocarriers. It will also establish novel concepts like multi-targeting and multi-functional nanocarriers for the treatment of colonic disorders.

## Introduction

**Figure 1:**
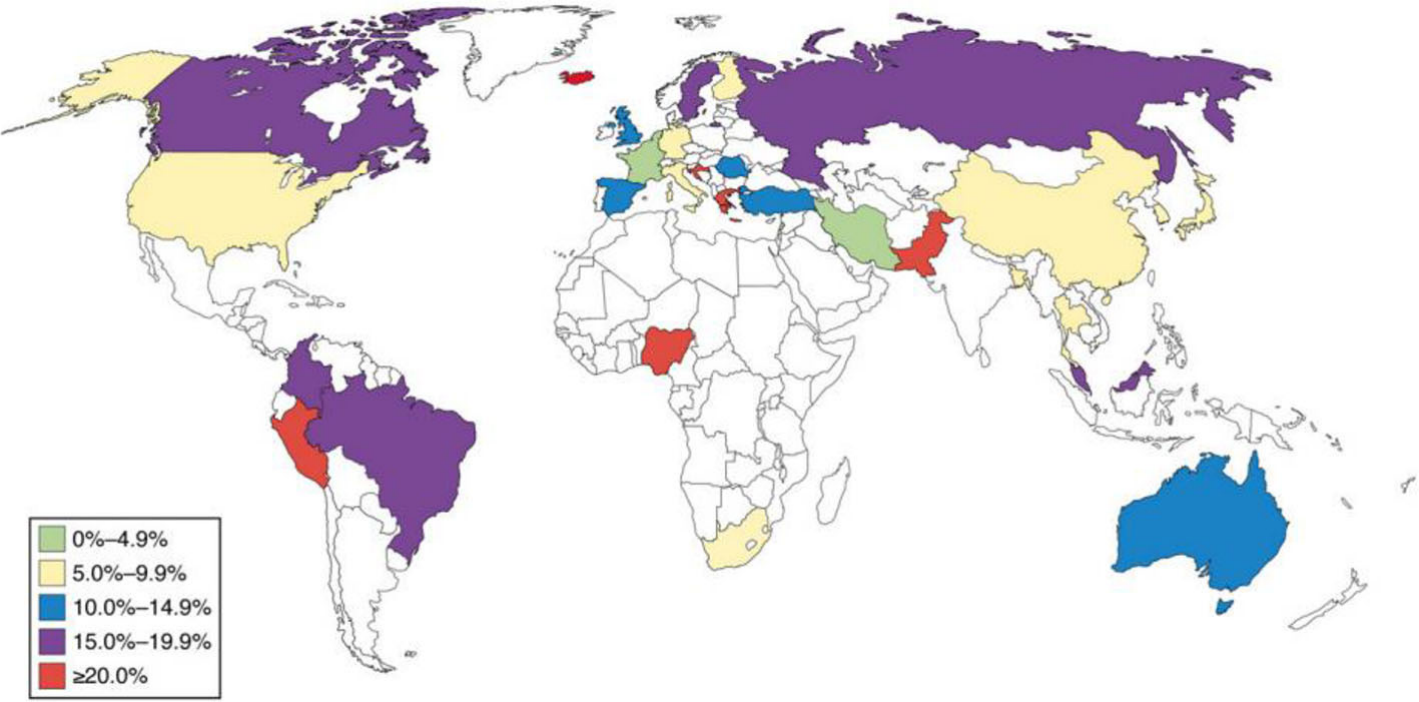
Global prevalence of intestinal bowel syndrome.

Inflammatory bowel disease (IBD) includes broad class of diseases like ulcerative colitis (UC) and Crohn’s disease (CD) which embark in young adulthood and prevail throughout the life (Cosnes et al., 2011). The etiology of these diseases is unknown, even though considerable progress has been made in comprehending these diseases. The incidence of these diseases is increasing worldwide, and despite of advances in therapeutics, the diseases still remain incurable (Reddy, 1999). A pictorial representation of prevalence of Intestinal bowel syndrome worldwide is shown in **Figure 1** (Lovell and Ford, 2012).

A heavy economic and health burden is placed on IBD affected populations as the disease reduces quality of life and amplitude for work and increases disability of an individual considerably. Takeuchi et al. (2006) discussed about most prescribed anti-rheumatic drugs, conventional NSAIDs, who have shown their efficiency as anti-inflammatory analgesics for IBD. Study conducted by Takeuchi and colleagues (2006), in their study, assessed the effect of these drugs in IBD affected individuals. Initially, individuals identified with inactive UC and CD, were administered with acetaminophen, or other NSAIDs and a non-NSAID analgesic. Intestinal inflammation was then quantified to examine the relapse mechanism. The patients were closely monitored for clinical relapse. As a part of the study stool samples collected from participants were evaluated for calprotectin measurement. Based on the observations made, the researchers categorized patients on the basis of their demographic and clinical details and clinical relapse induced by various NSAIDS. The detailed analysis of the data is mentioned in **Table 1**.

**Table 1:** Demographic and clinical details of patients (Takeuchi et al. 2006).

	Acetaminophen	Naproxen	Diclofenac	Indomethacin	Acetaminophen	Naproxen	Nabumetone
**Male/female**	12/14	14/18	19/10	9/13	13/7	9/11	8/12
**Age, median**	37 (26–24)	40 (20–70)	33 (20–68)	38 (24–70)	36 (21–66)	40 (20–58)	41 (26–69)
**Ulcerative cells**							
**Total**	5	4	5	7	3	1	4
**Left-sided**	5	5	7	6	6	9	7
**Proctitis**	6	3	5	2	1	2	3
**Crohn’s disease**							
**Small bowel**	4	11	7	5	7	4	4
**Colonic**	6	9	5	3	3	4	2
**Treatment**							
**Mesalamine**	17	19	16	15	15	15	17
**Azathioprine**	7	6	3	4	3	6	3
**Corticosteroids**	2	1	1	0	1	2	0

From this study, it was found early relapse on nimesulide, aspirin, or acetaminophen was not encountered with any patient. Also, nearly 20% of patients experienced relapse on naproxen and nabumetone. The research group inferred that ingestion of NSAID is linked with early relapse of inactive inflammatory bowel disease, owing to the COX enzymes and their dual inhibition. In another research findings, it was observed that any region of the gastrointestinal tract can be affected by CD inflammation (Podolsky, 2002) and it affects the ileum and colon with discontinuous inflammation. Increased mucus production, leading to the development of thick mucus layer in ulcerated areas is observed in CD. This makes mucoadhesion, a novel strategy for drug delivery systems in colitis (Antoni et al., 2014).

There have been recent advances in the field of research related to IBD. Colombel and Mahadevan (2017), discussed about the changing paradigms with regard to IBD. The study revealed a constant increase in the number of people being affected by IBD. Estimations presume that, since 2011, the number of patients suffering from IBD has increased by 200,000 in US. Currently, about 1.6 million Americans have IBD and 70,000 new cases of IBD are diagnosed each year. Kaplan and Ng (2017) identified westernization of diets and environments, as the primary cause for the rise in the prevalence of IBD. Such modification in the diet affects the intestinal microbiome and increases the risk of IBD in genetically susceptible individuals. In terms of the genetic approach, analysis of Paneth cell phenotypes holds significance in the genetics of IBD since the discovery of *NOD2* in 2001. The phenotype of the Paneth cell facilitates detection of the risk of disease progression and response to biologics for the IBD patients (Frank et al., 2007). Usually in response to the risk alleles for CD, ATG16L1 and NOD2, the phenotype of the Paneth cell gets altered (Colombel and Mahadevan, 2017). In the recent years another developing field that has gathered immense attraction is the role of microbiome in IBD. In this regard Sartor and Wu (2017) in their study identified the role of gut bacteria, fungi, and viruses in mediating mucosal homeostasis, via their composite genes and metabolic products. The concept of “dysbiosis” and emergence of IBD has been now well established. Condition of dysbiosis leads to alterations in the metagenome and metabolome profiles causing inflammation and effector immune responses that in turn mediate inflammatory bowel diseases (IBD) in humans. Cader and Kaser (2013), revealed the importance of the intestinal microbiota in ensuring the proper development and function of the immune system which appears to have evolutionarily coevolved. Immune cells are the key players in maintaining the intestinal homeostasis. Thus, alteration in their function or emergence of any imbalance may lead to IBD. The study by Sartor and Wu (2017) thus proposed the possibility of utilizing adjuncts or immunosuppressive drugs and dietary management to engineer the microbiota community structure or function in the intestinal environment for treating patients with IBD.

## Critical Aspects of Drug Delivery and Dosage Form Developments

For all drug developments, pharmacokinetic profiling by ADMET (absorption, distribution, metabolism, excretion, and toxicity) is a significant aspect (Rezvanfar et al., 2012). Researchers have prominently mentioned that, if the ADMET properties are poor, candidate drug development process might be ceased, either in early phase of drug discovery, or during the process of drug development. During designing, synthesis, and development of new drugs, the optimum pharmacological effect is characterized for optimum absorption, distribution, metabolism, and excretion (ADME) along with minimal toxicity (T) and enhanced selectivity. In actuality, the resultant favorable outcomes of the given drug rests on its optimum ADMET properties. Tiwari et al. (2012) have illustrated the role of drug delivery in administration of a drug to achieve the desired therapeutic effect and attain aforementioned desired attributes. It is essential that developed dosage form should show minimal side effects. Conventional dosage forms when delivered by their specified routes of administration seldom provide localized targeted effect. Some of the limitations, drawbacks associated with conventional formulations can be resolved using carrier-based delivery systems like liposomes, proliposomes, microspheres, gels, prodrugs, cyclodextrins inclusion complexes.

Buchman (2001) concluded that the current challenges in drug delivery approaches, is inability in preventing and achieving reduction of drug-related side effects. Mild to severe adverse drug reactions, including mortality, are most prominent effects observed in the current treatment of IBD. Long and short term side effects, like hypertension, osteoporosis, and depression are shown with the increased use of corticosteroids (Tiwari et al., 2012). Also, attempted treatment with immunosuppressive agents increases the risk of susceptibility to infections and malignoma (Cunliffe and Scott, 2002; Mason and Siegel, 2013). Consequently, the nursing of IBD demands a right equilibrium between better therapeutic efficiency of drugs and the possibility of adverse drug reactions. Evaluation of benefit to risk ratio is very essential as ADRs may weaken the life quality related to health and may therefore prevent successful treatment of the disorder. To improvise therapeutic efficiency, and to reduce the adverse drug reactions, selective drug accumulation inside the colon at the inflamed sites can be done with the help of nanocarriers based delivery systems supplemented and coupled with active targeting approaches like ligands (Mane and Muro, 2012). An expanding number of ligands for targeted drug delivery are studied for drug approaches related to drug targeting specific for colon. Recent advances in ligand anchored therapy and its significance on the development of targeted nanocarriers will be explained in this review. It will also introduce novel concepts like multifunctional nanocarriers and multi-targeting in the treatment of colonic disorders.

According to Subal (2005), traditional delivery of drug meant predictable absorption of a drug or a chemical from the site of injection or gut. Until now, the focus of drug delivery has been emphasized on maintaining zero order in receiver’s body throughout the day (Subal, 2005). However, living organisms demand drugs at different amounts, in accordance with the circadian rhythms in order to minimize risks and maximize the required effects (Hrushesky, 2001).

Hilt and Peppas (2005) observed that improving the drug delivery systems can lead the annual drug delivery sales to increase tremendously. Common routes in traditional drug delivery have certain advantages and disadvantages associated with them. The routes are pulmonary, injection, transdermal, and oral (Hilt and Peppas, 2005). Except for direct injection for vein or muscle tissue, all these other routes have cellular layers and acts as an obstacle for systemic circulation. A significant increase in therapeutic activity of a drug can be observed by controlled release in drug delivery. However, facilitated drug delivery is not possible with such traditional drug delivery methods (Peppas, 2004).

Overall, it is discerned that the developed dosage form should show minimal side effects. Primarily, prevention and achieving a reduction of drug-related side effects are found to be the current challenges. Mortality, hypertension, osteoporosis, and depression and increased risk of susceptibility to infections and malignoma are few of the limitations of conventional dosage forms elucidated. To prevail over the above discussed limitations related to the conventional dosage forms, a strong need for the development of non-conventional dosage is required (Malayandi et al., 2014). According to Brahamanker and Jaiswal (2004), there are two solutions to overcome such situation: Development of better and novel drugs having long half-life and higher therapeutic indices, and efficient use of existing drugs through utilizing targeted drug delivery systems. Since the past three decades, controlled dosage drug delivery systems are developed due to their advantages. Skelly and Bar (1987), thus pointed at achieving more predictable and increased bioavailability of drugs.

## Colonic Drug Delivery and its Physiological Landscape

Owing to the unavailability of appropriate therapeutics for treatment of IBD, corticosteroids till date poses as the main clinical IBD therapeutics in spite of having significant side effects. Heavy corticosteroid use may even result in the failure of IBD treatment leading to respective surgery (Wang et al., 2018). Such drugs are administered orally. However, oral release of drugs requires designing of appropriate delivery systems that can remain intact throughout gastric residence and along the small intestinal transit, and also ensures that the drug is released post entry into the large intestine, after responding to extrinsic targets (Ensign et al., 2012). For this reason, a number of approaches, each exploiting different physiological parameters such as microflora distribution, pH, intraluminal pressure and, finally, residence/transit time have been attempted. However, according to Galindo-Rodriguez et al. (2005), most orally administered drugs undergo direct transit through the colon and are not retained, thus, mucoadhesion attempts to enhance the time of residence of the particles in the gastrointestinal tract. Mucoadhesive polymeric systems, mucoadhesive pH-responsive systems, mucus penetrating systems are some of the novel approaches allow targeted delivery into the gastrointestinal tract (Ensign et al., 2012). CD is primarily characterized by increased mucus production, leading to the development of thick mucus layer in ulcerated areas. This phenomenon allows implementation of mucoadhesion as a novel strategy for drug delivery systems in colitis (Antoni et al., 2014).

**Figure 2:**
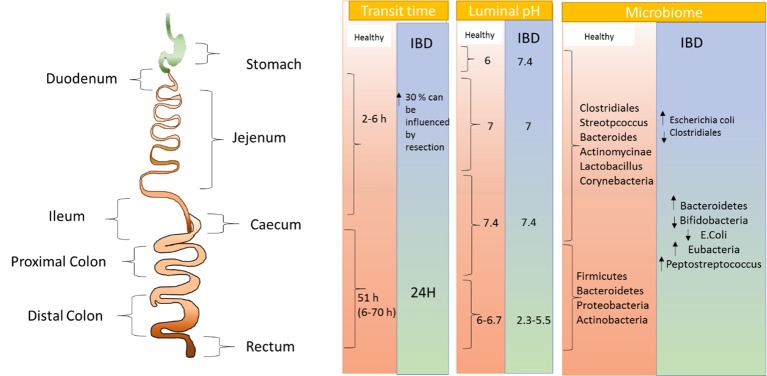
Pictorial representation of physiological as well as microbial changes to the gastrointestinal tract in inflammatory bowel disease.

Maneuvering optimum drug delivery to desired site of action i.e. colon following oral ingestion of drug depends on a number of physiological factors as shown in **Figure 2** (Hua et al., 2015). Considering transit time of the formulation which subject to variations due to number of factors like food, patient physiology, it is significant to ensure drug delivery to the action site (Asghar and Chandran, 2006). Four hours is generally accepted as transit time of small intestine, wherein, the individual time lies within 4 to 6 hours, which varies from individuals with the range of 2 to 6 hours. On the other hand, the transit time of colon can significantly vary between 6 to 70 hours (Hua et al., 2015). Gender also plays an important role in confounding the transit time through the GI tract, where females are found to have significantly longer colonic transit times (Buhmann et al., 2007). Comprehending the reason why the therapies are delayed, Bratten and Jones (2006) assessed the pH difference along the colon. They suggested that as the stomach environment is highly acidic, it is found that the duodenum has a pH 6, which then elevates to pH 7.4 at the terminal ileum. However, pH may vary among individuals depending on factors like food and water intake along with metabolism by microbes (Ibekwe et al., 2008). Additional to influencing pH, drug delivery to the colon may be interrupted by the environment of the GI tract. This, aspect, is supported by study conducted by Fatouros and Mullertz (2008). The group found that after intake of food, digestive enzymes, bile salts, and volume of free fluid get altered significantly. The probability of drugs being taken up by cells is also influenced by the viscosity of mucus layer (Keely et al., 2011).

To attain the required therapeutic effect of any drug in blood and plasma and to maintain its constancy during the treatment, an ideal dosage regimen for the drug is required. This can be attained through the administration of conventional dosage at a pre-estimated frequency. The half-life of a drug decides the frequency of administration or dose interval of any drugs. However, in many cases, interval of dosing is shorter than the drug’s half-life, which leads to a number of limitations relevant to such dosage forms. Brahamanker and Jaiswal (2004) listed the limitations of using conventional drug delivery as poor compliance of patients that is for a drug where frequent administration is necessary increased chances of missing the dose of a drug with relatively shorter half-life. Sometimes, a representative peak plasma concentration time profile is obtained, which makes attaining steady conditions difficult (Brahamanker and Jaiswal, 2004). They also listed that the unpreventable variation in the drug concentration might lead to underdosage of overdosage beyond the therapeutic range.

A large amount of research has thus been made to explore new arenas for colonic drug delivery for the patients suffering from IBD. Teruel et al. (2018) designed a novel oral colon drug delivery device. The nano device consisted on magnetic mesoporous silica microparticles loaded with safranin O (**S1**) or with hydrocortisone (**S2**) and functionalized in the external surface with a bulky azo derivative covalently grafted through urea bonds. The efficacy of the device in treating IBD was assessed *in vivo* in a 2,4,6-trinitrobenzenesulfonic acid solution (TNBS) colitis induced rat model. At neutral pH both S1 and S2 remained capped, however on exposure to a reducing environment such as in the presence of sodium dithionite, payload release enhanced significantly. IBD therapy proposed by Wang et al. (2018) involved a newly designed drug-delivery system that delivered an anti-inflammatory corticosteroid called dexamethasone (Dex). Study results revealed that in presence of esterase, 10% Dex loaded, PPNP-Dex (polymers self-assembled nanoparticle- dexamethasone) exhibited responsive release behavior. In addition, the radical scavenging activity of PPNP-DEX at the inflammation sites enhanced the drug retention rates in mice with colitis. Thus, PPNP poses immense potential as a drug-delivery platform for IBD therapy. Zhang et al. (2015) also utilized Dex for treatment of IBD. IT-hydrogel microfibers loaded with Dex used in the study were not only stable but also released only after enzymatic digestion. The IT-hydrogel combined with Dex exhibited preferential adhesion to inflamed epithelial surfaces both *in vitro* and *in vivo* models. IT-hydrogel drug delivery platform also holds promise in future as a targeted enema-based therapy in patients with colonic IBD. Oral drug delivery being the most convenient method for colon-specific targeting and the treatment of IBD, Qiao et al. (2017) examined the potential of amphiphilic curcumin polymer (PCur) as a therapeutic treatment for IBD in mouse model. The suitability of PCur as an active drug to treat IBD results from its sufficient solubility, nano-scaled size, and close to the neutral surface potential. Additionally, PCur in its active form exhibits preferential accumulation in the inflamed regions of the gut and limited drug release and at the physiological pH of the gastrointestinal tract (GIT). However, within the colon of the dextran sodium sulfate (DSS) induced murine model of IBD, orally administered PCur restricted the inflammatory progression in the colon and thus rendered protection to the mice from IBD. Thus, PCur conjugate could pose as a potentially candidate for colon-specific treatment of IBD (Kreuter et al., 1989).

Chatterjee, Bong, and Zhang, advocated the ability of nanotechnology to attach ligands to the carriers. Guimarães and Ré (2011), in their study used lipid nanoparticles as carriers for cosmetic ingredients. The first generation of lipid nanoparticles, called SLC (Solid Lipid Nanoparticle) replaced the liquid part of an emulsion by a solid part resulting in the matrix of lipid at body temperature and room temperature. A second generation, called NLC (nanostructured lipid carriers), were manufactured with the blend of liquid and solid lipids. Garg et al. (2016) utilized NLC for effective transdermal delivery of methotrexate (MTX). NLCs not only exhibited better skin permeation with higher permeation flux but also stood high on the safety potential parameters. The scope of vitamin D3 in treatment of IBD was assessed by Zai et al. (2018). Vitamin D3 in its active form has been reported to exert significant positive effect on the suppression of IBD. However, realization of the same requires implementation of high dosage of vitamin D3. Nanostructured lipid carrier (NLC) serves as an effective drug delivery tool. Zai et al. (2018) designed an orally administered colonic delivery model wherein nanostructured lipid carrier (NLC) was used for encapsulation of 1,25(OH)2D3. The NLC-D3 model exhibited successful suppression of the multiple symptoms of colitis induced by DSS and thus poses as a promising alternative treatment for IBD therapy. A recent study by Garg et al. (2019) however, highlighted on the absence of appropriately characterized intestinal vitamin D receptor (VDR) for patients with IBD. Future research is thus required to design strategies that would upregulate the expression of VDR thereby facilitating therapeutic approaches that implements vitamin D for treatment of IBD. Shi et al. (2018) conducted *in vitro* and *in vivo* study using pH-sensitive and colon-targeting P(LE-IA-MEG) hydrogel microspheres for UC therapy. Anti-inflammatory drug hydrocortisone sodium succinate (HSS) was combined with pH-sensitive (PLE-IA-MEG) hydrogel microspheres (HMSs) as the drug carrier for the treatment of UC. Results revealed that compared to HSS alone, HSS-HMSs rendered enhanced therapeutic effects on mice with experimental UC. Kumar et al. (2015) in their study concluded that clinical and non-clinical stages of drug development should include analysis of thermokinetic information to define and comprehend the toxicological and pharmacological characteristics of candidate ligand (Ghosh et al., 2008).

## Limitations of Traditional Drug Delivery Methods In Colon Disorders

Drwal and Griffth (2013) proposed the amalgamation of molecular modeling methods based on ligand- and structures as an approach to virtually screen and described various procedures to integrate ligand based and structure based methods for distinguishing appropriate ligand for targeting. The group mentioned of hybrid, sequential, or parallel approaches. When structural information about the target is inadequate, then biological and chemical properties of such ligands are analyzed (Drwal and Griffth (2013)).

The importance of intestinal microbe in colon physiology is now appreciated as it hosts over more than 500 bacterial species, the estimated number of bacterial species to be close to 2,000 (Sartor, 2008). The microbiota plays a significant part in digestion and intestinal health, metabolism of proteins, carbohydrates, and fatty acids (Hua et al., 2015). There are certain synthetic polymers for example azopolymers and natural gums like guar, locust, pectin, which are exclusively degraded by enzymes secreted by microflora of colon. These polymers have been studied as carriers extensively for colon targeting because of their unique properties. Studies suggest that there is an immense variability in microbial population of colon of healthy and diseased individuals. Owing to the complexity of changes in physiology associated with colon disease, the efficacy of orally administered microbial enzyme triggered polymer based formulations is severely affected. The physiological factors that pose as challenges in dosage form design are interrelated and dynamic and thus remain an important challenge in drug designing (Sartor, 2008). Therefore, oral drug delivery can be affected by the physiological variables of gastrointestinal pathologies.

The review by Ratnaparkhi et al. (2013) compared the various approaches like pH, time dependent systems, prodrugs, and microbial triggered systems for Colon Specific Drug Delivery. They found various limitations like extensive steps of manufacturing, effect on colonic performance by resident microflora through metabolic drug digestion. Also, bioavailability of drugs was found to be low due to potentially binding of drug in a nonspecific way to dietary residues, intestinal secretions, mucus, or fecal matter. They found that if drug is not in an absorbable form, the solubility of the drug will get affected. Another important limitation of the pH sensitive coating technique is that the drug may start to dissolve irrespective of the target location and environment. The prodrug approach also has the limitations of non-versatility as the formulation relies on the functional group for chemical linkages on the drug moiety.

The delivery of specific drugs to colon justifies the scientific rationale through the concept of targeting. To comprehend and achieve the required goal of targeting the delivery to colon, various approaches are being researched. However, the available approaches have their limitations and challenges. At present, the need is to recognize the approximate approaches which can lead to safe and effective drug delivery.

## Need of Ligand Based Colon Targeted Drug Delivery

UC and CD are included in the category of chronic relapsing gastrointestinal (GI) diseases (Podolsky, 2002). Hua et al., 2015 devised a strategy for targeted drug delivery to unhealthy colonic tissues which has been advantageous for high levels of reactive oxygen species (ROS) formed at the location of intestinal inflammation. In the study conducted by Hua et al., 2015, inhibition of production of TNF-α (tissue necrosis factor) and selective biodistribution of siRNA (small interfering RNA) in ulcerative tissue was observed during the *in vitro* and *in vivo* and studies. Owing to galactose receptor-mediated endocytosis, cellular uptake of nanoparticles in activated macrophages was found to be significantly higher for GTC/TPP (galactosylated trimethyl chitosan/tripolyphosphate) nanoparticles.

As per a study contemplated by Naeem et al. (2018) colon-targeted delivery of cyclosporine A using the dual-functional Eudragit® FS30D/PLGA nanoparticles ameliorates murine experimental colitis was explored in the study. The study elucidated how colon-targeted oral nanoparticles have been perceived to emerge as an ideal, effective, and safe therapy for UC owing to their ability to accumulate in inflamed colonic mucosa selectively. While a study by Chauhan et al. (2010) evaluated colon targeted drug delivery system. The researcher asserted in the study that prednisolone is an anti-inflammatory drug. Majorly, it is used clinically for the oral administration in the treatment of diseases of colon whereby high local concentration can be achieved while minimizing the side effects that occur because of the release of drugs on the upper GIT or unnecessary systemic absorption. While, as per a review asserted by Aggarwal et al. (2011), the researchers explored and highlighted the recent trends in the colon targeted drug delivery system. The study examined the Colon Targeted Drug Delivery System (CTDDS).

Moreover, there are various mechanisms which are adopted for the drug release in this area. These have a coating with pH-sensitive polymers, e.g. Eudragit®S100, Eudragit® L100; biodegradable polymer like polymers which are sensitive to colonic bacteria; and polymer which selectively sticks to the colonic mucosa, e.g. polycarbophil or polyethene. The study further explored the need for CTDDS.

Majorly, the colon-specific drug delivery is critical for the treatment of diseases of the colon, such as colon cancer, irritable bowel syndrome, amoebiasis, and inflammatory bowel disease. While, oral route for the drug administration is discerned to be the most convenient way due to its simplicity, being noninvasive method and effectiveness. Shruti (2007), addressed the advantage of CTDDS over the conventional drug. As per the study, the colon has less peptidase activity so peptides, oral insulin vaccines, growth hormone can be delivered through this route while Akhil (2011) asserted that due to locality targeting in CTDDS small drug quantities are required. Additionally, since the dosage frequency is reduced, hence it has a lower cost of expensive drugs. While there are several limitations also pertaining to the colon targeting drug delivery system. Chiefly, there are multiple manufacturing steps and incomplete release rate. Also, as a per research manifested by Kaur et al. (2013), non-availability of an appropriate dissolution testing method to evaluate the dosage form *in vitro*. According to a study by Hua et al. (2015), the active targeting approaches using the ligands coupled to the surface of nano-delivery systems was perceived to increase the therapeutic efficiency. Moreover, it is discerned to reduce the adverse reactions, by further improving the selective drug accumulation at inflamed sites within the colon. While according to Mane and Muro (2012) the biodistribution and cellular uptake of polystyrene nanoparticles coated with anti-ICAM-1 antibodies was evaluated. According to Nayak et al. (2018), the current status of drug delivery technologies was explored and highlighted. The study thereby asserted examples of newer devices with tremendously improved therapeutic potential including oral controlled release systems, liposomes, fast dispersing dosage forms, taste-masking systems, aerosols, transdermal patches, and site-specific delivery systems. As per a study contemplated by Parhi et al. (2012), the emerging approach of nanotechnology-based combinational drug delivery was studied (Han et al. 2012). The study asserted that the treatment is becoming more popular because it generates synergistic anticancer effects, reduces individual drug-related toxicity and further suppresses multi-drug resistance through different mechanisms of action.

A barrier to treatment of colonic cancer is high drug toxicity because drug dosage can be limited by the side effects and this phenomenon is best exemplified by cytotoxic cancer drugs (Malam et al., 2009). Thus, the drug therapeutic index can be improved with the use of ligand–drug–nanocarrier complexes according to the following equation:

$$\mathrm{Tuherapeutic Index}=\frac{\mathrm{Maximum nontoxic dose}}{\mathrm{Minimum effective dose}}$$

**Figure 3:**
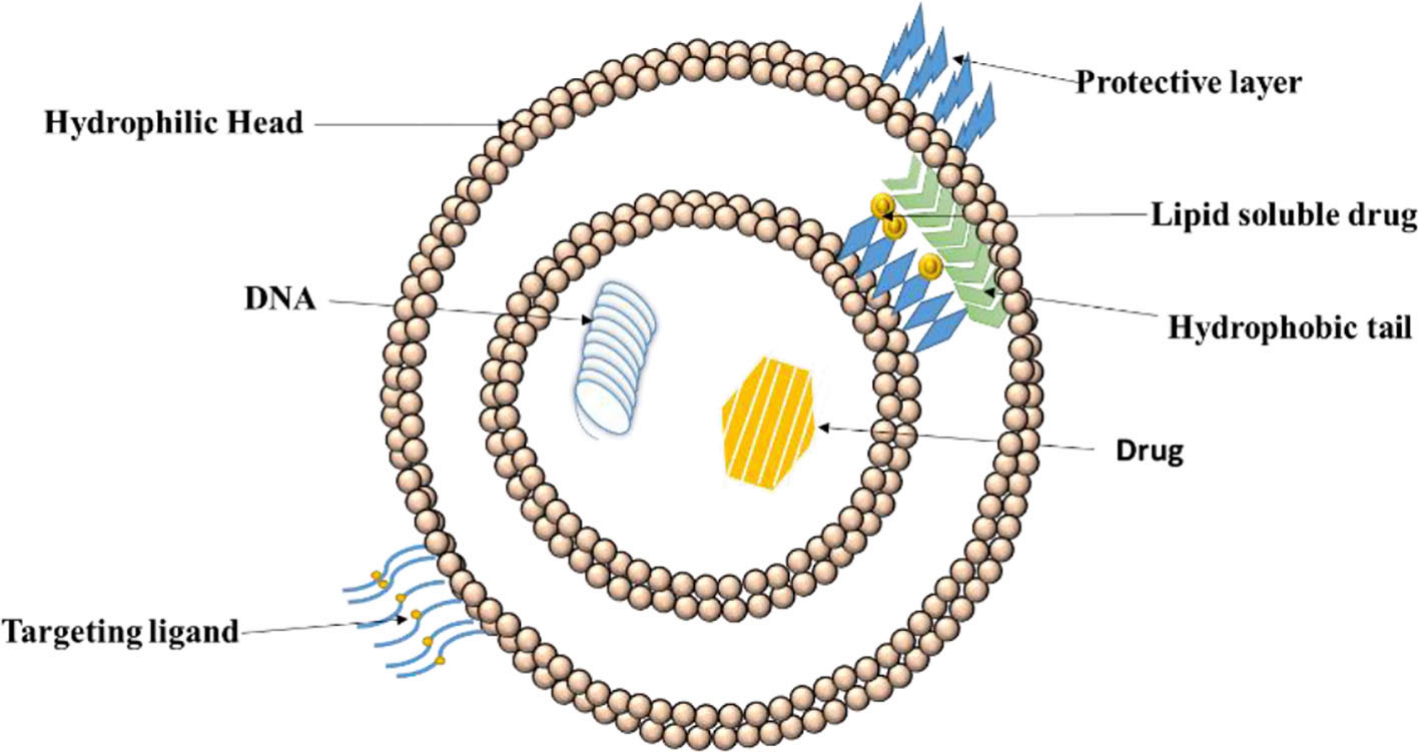
Positioning of ligand on liposomes for drug delivery. Active drug targeting achieved by conjugation of specific ligands to the liposomes specific to target cell receptor leading to efficient drug internalization.

Vega-Villa et al. (2008) stated that the high specificity, along with high selectivity of the ligand-complex improvises the quantity of drug to be delivered to select tissues and lessens the amount of drug at sites that are not wanted. Thus, lesser quantity of systematic drug requires to be managed to assure an adequate concentration at action site and also lowers the minimum effective dosage. Additionally, with the help of ligands, as lesser drug is available at non-target sites, the non-toxicity is ensured. Kaur et al. (2008) also found that the overall effect after the use of ligands drastically decreased toxicity and adverse drug reactions. **Figure 3** depicts functionalization and positioning of ligands for active targeting with help of liposomes.

## Oral Nano-Delivery System Approaches for Colon Targeted Drug Delivery

As asserted by Steichen et al. (2013), nanoparticles are colloidal carriers with dimensions on the nanoscale (10^−9^ m). Owing to their size, malleable composition, functionality of surface, and stability which provides unique opportunities for the interaction and targeting, nanoparticles are significantly attractive for their properties as a drug carrier (Park et al., 2009).

A polymer, poly (called ethylene glycol) [PEG; (CH2CH2O)n], is conjugated to the drug carrier (Hoffman, 2008). This process is called as PEGylation. At Rutgers University in the 1960s, it was identified to be a significant method to evade opsonization of large narrow carriers developed. The clearance of the drug by mononuclear phagocytic system is evaded by reducing opsonization of liposomes by PEGylation, thus increasing the half-life of circulation. Such a problem in the development of liposomes useful for treatment is presented by opsonization. Therefore, most research involves PEG-coated or PEGylated liposomes. The effect of PEG liposomal doxorubicin (Doxil1) was investigated in a male mouse tumor model inoculated with either colon cancer (C26) cells or their doxorubicin-resistant (MDR) subclone by Ogawara et al. (2009). This leads to overexpression of P-gp efflux pumps. The results of this study depicted the anti-tumor effects of PEG liposomal doxorubicin on doxorubicin-resistant and non-doxorubicin-resistant C26 cells.

**Figure 4:**
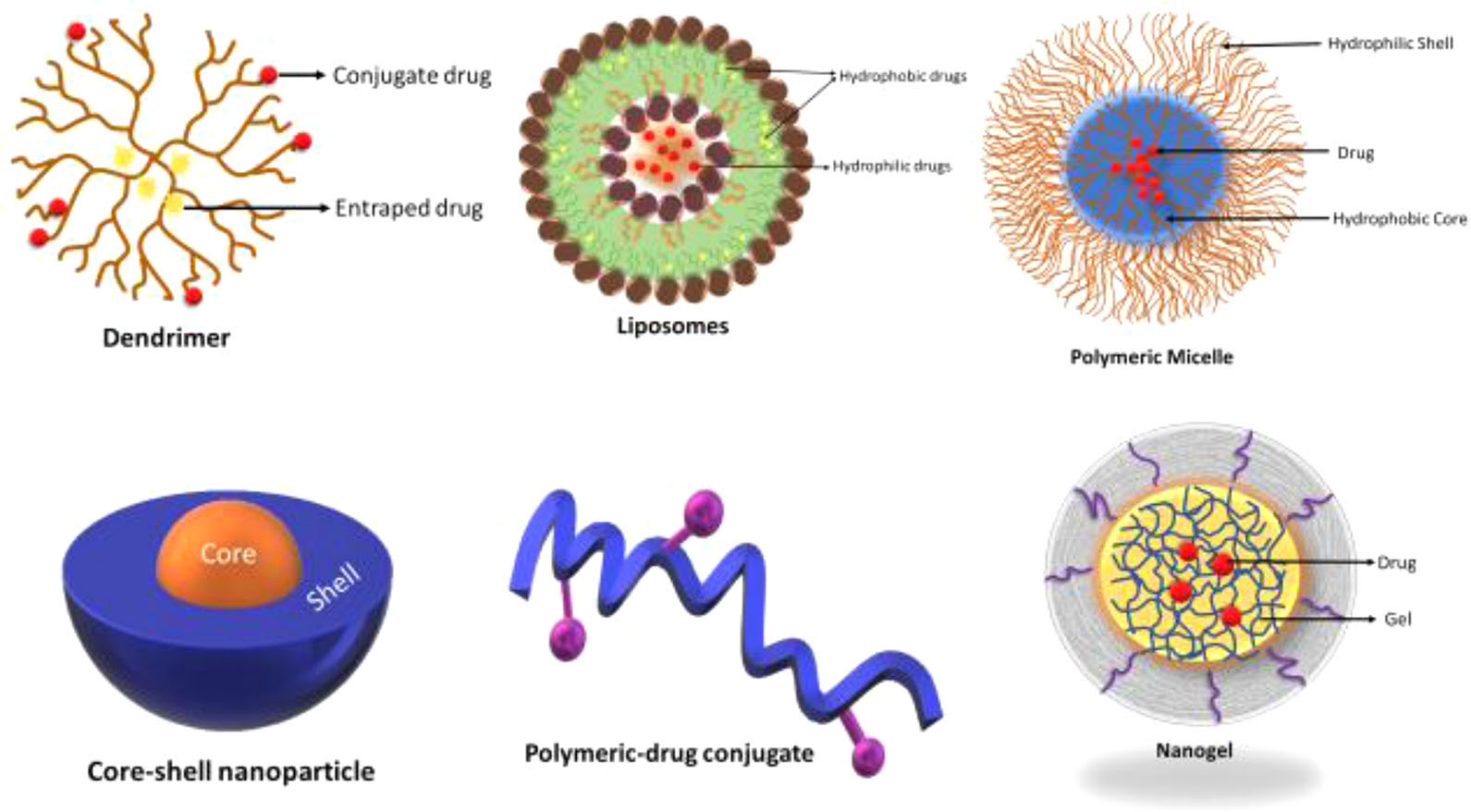
Several examples of pH-sensitive nanocarrier platforms.

The cancerous tumor mass, at early, as well as at the later stages, have a pH that is lower than that of extracellular area (pHe) surrounding normal tissue (pH about 7.4). This level ideally varies for different typ4es of cancer like lung cancer, breast cancer, and gastrointestinal cancer depending on the type, anatomical location, and size of the cancer tumor (Volk et al., 1993). Nevertheless, as suggested by Criscione et al. (2009), the pH environment occasionally increases in a tumor due to tumor necrosis that occurs in mass. The invagination in the plasma membrane engulfs the drugs which then get internalized by endocytic update into the cell (Mellman et al., 1985). Several examples of pH sensitive nanoparticle platforms are illustrated in **Figure 4**.

## Nanocarriers and Their Active-Targeting Ligands

The delivery of drugs to a target with the use of certain interactions like antigen–antibody and ligand–receptor binding at the target site is referred to as active targeting where there is a requirement of pharmacological activities of a drug. As another option, signals like magnetic fields and thermal energy might be utilized for active targeting by applying it externally to the target. Peripherally conjugated targeting moieties can be utilized to upgrade the delivery of nanoparticles. For cellular update, the targeting moieties are significant. Modifications at the surface in order to increase hydrophilicity can help in masking of the nano vectors from the reticuloendothelial system. This leads to an increase in circulation time and alters the pharmacokinetics of the active agents (Liechty and Peppas, 2012).

The study by Hinterdorfer et al., 2002, conducted to study the surface attachments of ligands asserts that when observed with atomic force microscopy, attachments of ligands and receptors is extremely tight. The binding of ligand to a drug might lead to activation or inactivation of a receptor and its activation which might, in turn, increase or decrease particular cell function. Each of the drug-bound ligands may interact with multiple receptor subtypes. Liechty and Peppas (2012) mentioned that many different nanoparticles, with their varied constitution and structures, helps in fine tuning of the specific applications and targets. Targeted drug delivery applications commonly use the structures like micelles, liposomes, nanospheres, dendrimers, and nanocapsules. Some of the most commonly used active targeting ligands for tumor therapy comprises of antibodies, folates, aptamers, transferrin, and peptides. Some examples of active targeting with the help of targeted drug delivery systems, with their respective targets and findings have been explained in **Table 2** below.

**Table 2:** Few common examples of active drug targeting with drug delivery systems.

Ligand/receptor	Study deliverance	Reference
Anti-CD74 antibody/CD74 receptors	Ligand attached to liposomes covalently(selective for malignant B lymphocytes)	Lundberg et al., 2004
TfR-targeting peptide HAIYPRH/TfR receptors	TfR peptide conjugation significantly improves the anticancer selectivity and efficacy of anticancer drug artemisinin	Oh et al., 2009
Folate/folate receptors	Folate receptors are overexpressed on cancer cells. Folate conjugated with liposomes containing doxorubicin for targeting on cancer with nanoparticles for targeted paclitaxel delivery	Kim and Martin, 2006
mBAFF/BAFF receptors	BAFF is the usual endogenous ligand for the BAFF receptor; mBAFF is a soluble BAFF mutant in which amino acids 217–224 are replaced by two glycine residues that can bind to BAFF receptors. PEGylated liposomes develop with mBAFF as targeting ligand and target certain B lymphoma cells *in vitro*	Zhang et al., 2008
Hyaluronic acid/hyaluronic receptors	HT 29 cancer cells overexpress hyaluronic receptors Hyaluronic acid incorporated in chitosan nanoparticles loaded with the anticancer drug 5-fluorouracil exhibited higher *in vitro* toxicity and cytotoxicity	Jain and Jain, 2008
Galactose/ASGP receptors	Hepatoma cells overexpress ASGP receptors. Dextran conjugated polymeric micelles used to target liver cancer showed better results *in vivo*.	Wu et al., 2009

**Figure 5:**
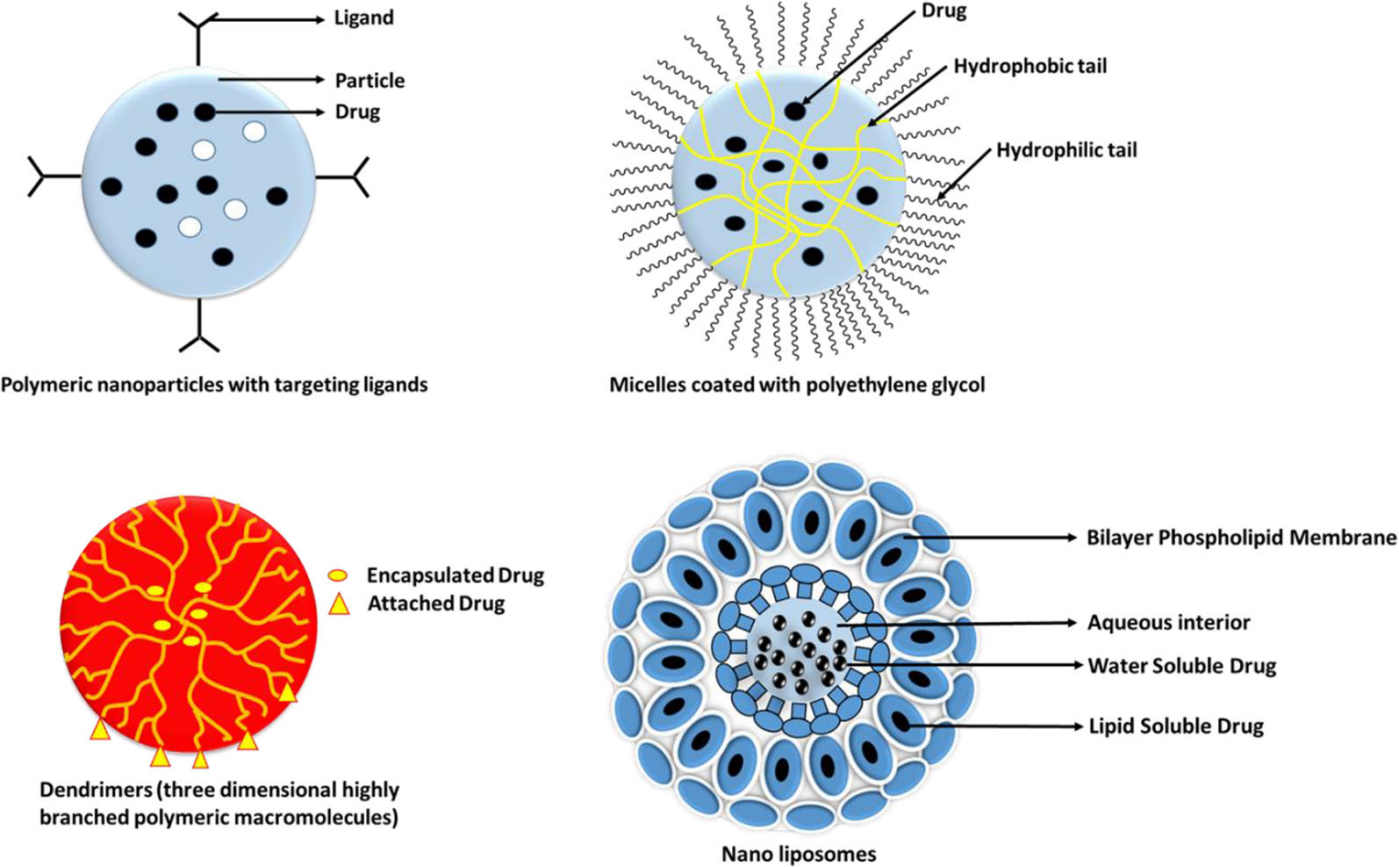
Types of nanocarriers for drug delivery.

The different types of ligand based nanocarriers for their drug delivery is shown below in **Figure 5** and different ligands have been discussed in the next section.

## Folate

The high affinity membrane folate binding protein, also known as the folate receptor (FR) was found by Gu et al. (2007). One such nanoparticle conjugated with a folate receptor was developed by Yoo and colleagues. They used the copolymers of poly ethylene glycol (PEG) and poly(D,L-lactic-co-glycolic acid) (PLGA) to create micelles. Biodegradation, after delivery of the carrier drug and the PEG is facilitated by PLGA, which increases the retention time of the particles. Yoo and Park (2004) examined these particles and assessed that the circulation time, cellular uptake, and decreased cardiotoxicity increases with these particles. This indicates the great capacity of targeting moiety to differentiate between tumor and healthy tissue. Also, increase in the cellular toxicity and uptake by the cells depicts the active internalization of the conjugate particle into the cytoplasm by the folate receptor (Yoo and Park, 2004). The study by Yi (2016) revealed the potential of folate receptors (FR) as a potential target for treatment of diseases associated with inflammation. Since the macrophages expresses large number of folate receptors and are the key contributors in inflammatory diseases, selective targeting of these FR-positive activated macrophages would pose as an efficient way to diagnose and treat inflammatory diseases. Similar attempts were made by Qi et al. (2015) and Yang et al. (2016) wherein active targeting of folate receptors in macrophages was employed to ensure site specific delivery of the drug at the inflammation site. For the drug delivery purpose, MTX conjugates of PAMAM dendrimers or biodegradable dextrans were utilized.

## Transferrin

Another example of ligand-receptor pair used to target tumors and for drug delivery is transferrin. It is a membrane glycoprotein, that along with its receptor, TfR, aids in uptake of iron by the cell (Ponka and Lok, 1999; Yoo and Park, 2004). Sahoo and colleagues studied and defined materials for drug delivery using both copolymerized PLGA and poly(vinyl alcohol) (PVA). Sahoo and Labhasetwar (2005), showed that transferrin-conjugated nanoparticles inhibit cellular proliferation and tumor growth while also increasing cell uptake. The efficacy of such conjugated nanoparticles mostly owes to their capacity of being taken up by receptor-mediated endocytosis (Sahoo et al., 2004). This also keeps in check, the amount of the drug that will be delivered to healthy cells (Sahoo and Labhasetwar, 2005).

## Aptamers

Short oligonucleotides of RNA or DNA having the potential to be folded into different conformations and are able to bind to ligands are c called aptamers (Gu et al., 2007). As devised by Wilson and Szostak (1999), a process to filter through vast RNA/DNA sequences to search new aptamers is called Systematic Evolution of Ligands by Exponential Amplification (SELEX). In research conducted by Dhar and colleagues (2008), nanoparticles conjugated with aptamers were used as drug delivery vehicles in cancer therapy as they showed significant drug delivery potential.

## Antibodies

The active targeting to different tumor types owes their properties to the specificity of antibodies. This is also due to their potential to identify and differentiate between cancerous and healthy cells, even among different types of cancers, but mostly in colorectal cancer (Dhar et al., 2008). As reported by Johnston et al. (2012), the antibody-conjugated particles, along with getting phagocytosed, also interacted with cancerous cells, thus acting as ideal vehicles for delivery of therapeutic drugs.

## Peptides

Steichen et al., 2013 proposed that peptides have the potential to target the drugs used for chemotherapy. Peptides, alike antibodies, shows the potential to be utilized to interrupt the tumor cells interaction that occurs on tumor cells and cease cellular proliferation. Peptides are also beneficial as they are much less costly and easier to manufacture than the antibodies (Brissette et al., 2006). Combinatorial type of phage library is widely used to screen protein ligands (Steichen et al., 2013). This technique leads to ligands having length from 10–15 amino acids and that are capable of identifying and binding with the tumors that have high affinity (Gu et al., 2007).

**Figure 6:**
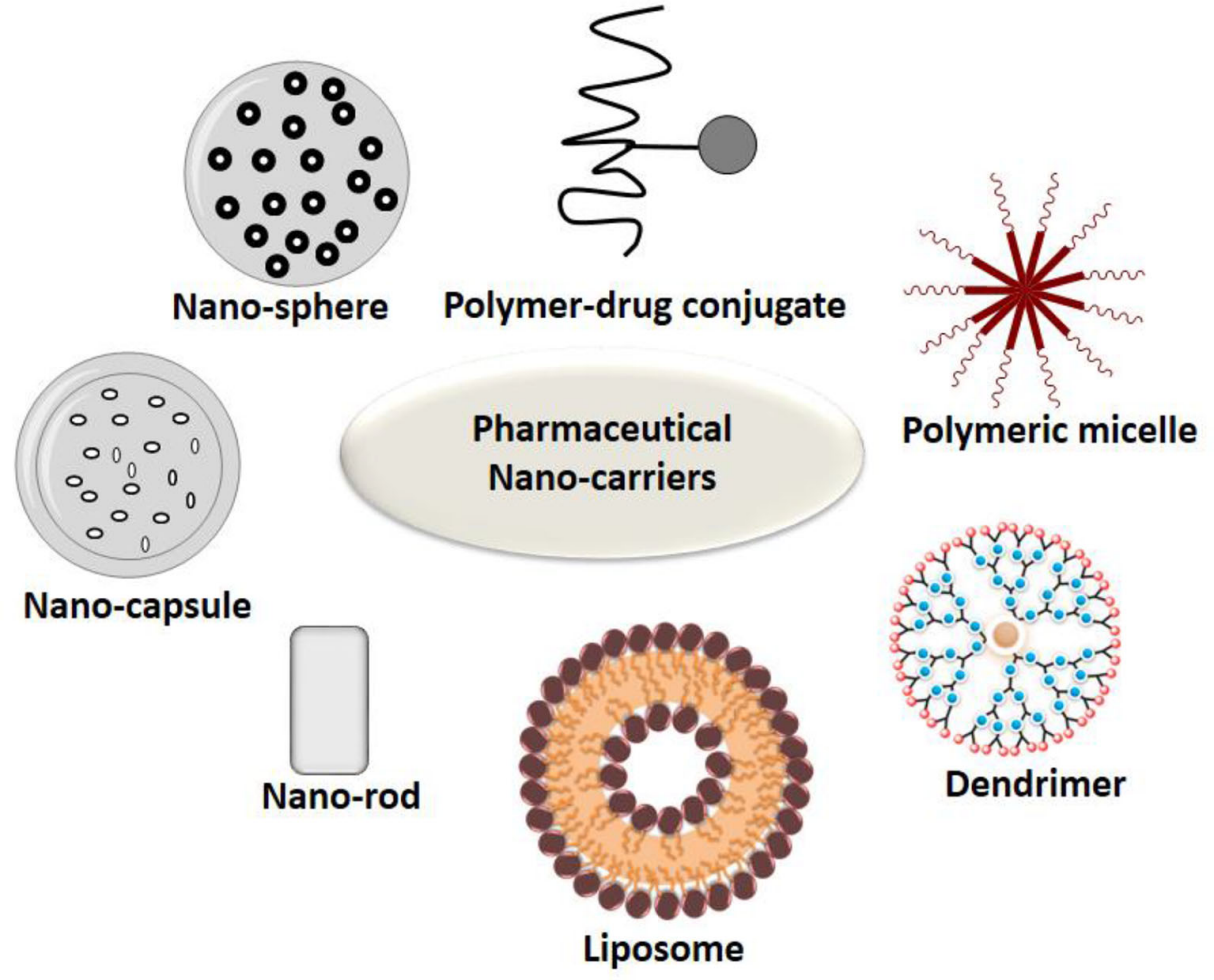
Structural design of a ligand-targeted drug conjugate.

**Figure 7:**
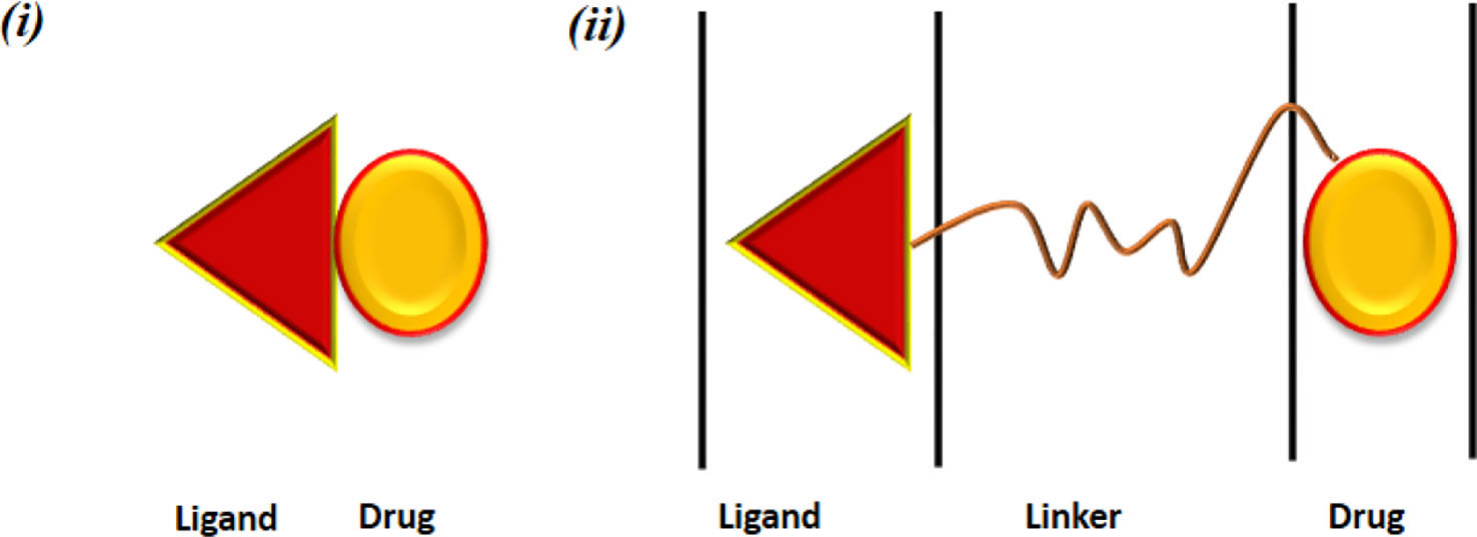
Pharmaceutical nanocarriers for drug targeting. Ligand-drug conjugate obtained by **(i)** direct linkage between the drug and the ligand and **(ii)** connected via a linker.

**Figure 6** Depicts structural design of a conjugate form of ligand and target drug. a significant requirement in this is the potential of carriers to circulate in the bloodstream for a long time period (Trapani et al., 2012). The schematics of positioning of a linker and target drug is depicted In **Figure 7**.

## Future Advances in Colon Targeted Drug Delivery

A new paradigm has been established in the pharmaceutical field by the advancement of new drug delivery systems using nanomers. Currently, a variety of nanoscale drug delivery systems are used in clinical trials and are already available commercially. For instance, Abelcet is used for the treatment of fungal infection, Doxil is used as an antineoplastic agent, abraxane to treat metastatic breast cancer, Emend as an antiemetic and so on (Mukherjee et al., 2014). However, as observed by Mukherjee et al., 2014 in the recent years, despite the impressive progress in this field, US-FDA has approved only a limited number of nanoformulations which have not even reached the pharma market. Chakroborty et al., 2013 reported the utmost biocompatibility of nanomaterials owing to efficacy of nanomaterials in the body varying from cytotoxicity to hypersensitivity. However, even when they have all these advantages, the clinical, regulatory, and toxicological aspects, are some of the toxicological concerns. In Application of Nanotechnology in Drug Delivery Kellenberger et al. (2004) used eight docking programs for single-ligand docking and database screening to differentiate random drug-like molecules from thymidine kinase, which is the known enzyme inhibitor. It was found that their properties are correlated as the tools show docking accuracy and successfully demonstrated inhibitors in a screening experiment. Zhou et al. (2007), in their comparative study, investigated both Standard Precision mode (SP) and Extra Precision mode (XP) of Glide for a diverse set of pharmaceutically relevant targets.

For local treatment of bowel diseases like CD and UC, colon targeted drug delivery (CTDD) is preferred (Podolsky, 2002). Glucocorticoids and other anti-inflammatory agents are currently used to treat diseases like Chronic colitis, namely UC, and CD are currently treated with glucocorticoids, and other anti-inflammatory agents (Steed et al., 1997). Thus, not only the necessary dose of the drug is reduced due to CTTD, but it also reduces the systemic risks and side effects (Hua, 2014). Systemic absorption is undesirable to most of the drugs against most oral regimes of administration. This colon targeted approach has thus been shown to reduce adverse drug reactions and increase therapeutic efficiency and allows utilization of new drugs with poor pharmacodynamic properties for oral delivery. The practical application of designing dosage forms which is effective as well as acceptable by humans further needs to be explored. However, from a commercial view, effective and dependable production of nanomaterials demands simplification of the drug delivery systems.

## Conclusion

Many novel technologies have been in the pipeline and are being developed to treat various diseases and therefore, nanotechnology is extensively used to develop nanocarriers for drug delivery. Conjugation of nanocarriers with a ligand favors targeted drug therapy approach (Petty and Lo, 2002). Thus, utilizing ligand targeted drug delivery system can transform the complete approach of drug therapy and elevate it to a better level in the future (Mukherjee et al., 2014).

However, the toxicity of the nanosize formulations should be considered and evident methodologies to assess minor as well as long term toxicity testing for the nanosized drug delivery systems. Numerous pathological conditions like Intestinal Bowel Diseases, cancers, and infections can be treated due to the development of nanoparticles (Vingerhoeds et al., 1994; Singh, 1999; Maruyama, 2002).

Nanoparticles are propitious candidates for drug delivery systems. Both the clinical and non-clinical evidence predominantly asserts the potential of nanoparticles in targeting colonic disorders Nanoparticles like folates, transferrins, aptamers, antibodies, and peptides have attractive properties like the low level of toxicity, biocompatibility, low clearance rates, and controlled drug release. Ligands with non-starch polysaccharide coatings, such as the COLAL-PRED® (prednisolone sodium metasulfobenzoate) system offers numerous advantages as an oral formulation for treatment of IBD. Eudragits® is another example of an approved pH dependent polymer for the treatment of IBD (Hua et al., 2015). Further study and evaluation of nanomaterials and its applications in humans still need to be assessed. Literature has been limited to ligands having short-term benefits.

Specificity of targeting have restricted effect owing to the current formulations (Hua et al., 2012) for targeting diseased colon tissue. Additionally, regardless of coverage to the diseased tissue of the colon during ligand-targeted drug delivery, the efficacy of drug uptake into the cells and tissues of the target area is not guaranteed. Thus, designing formulations with the help of nanotechnology is explored to further enhance the efficacy of therapeutics by targeting and up taking the drug inside the colon (Jani et al., 1989; Jani et al., 1990). *In vivo* studies can be further carried out to evaluate the effectiveness of the novel drug formulations that lead to phase 1 clinical trials. The duplicability of products after drug formation also needs to be evaluated in future.

## Author Contributions

PC contributed in conceptualization, planning, and writing of the manuscript. PC, MM, and SS contributed toward data collection, writing and editing of the manuscript.

## Conflict of Interest

The authors declare that the research was conducted in the absence of any commercial or financial relationships that could be construed as a potential conflict of interest.
